# Role of Glucagon-Like Peptide-1 Receptor Agonists in the Management of Non-Alcoholic Steatohepatitis: A Clinical Review Article

**DOI:** 10.7759/cureus.15141

**Published:** 2021-05-20

**Authors:** Haider Ghazanfar, Sameer D Kandhi, Iqra Nawaz, Nismat Javed, Minu C Abraham, Mohamed Farag, Jaydeep Mahasamudram, Vishwa B Patel, Faryal Altaf, Harish Patel

**Affiliations:** 1 Internal Medicine, BronxCare Health System, Bronx, USA; 2 Internal Medicine, Shifa College of Medicine, Shifa Tameer-E-Millat University, Islamabad, PAK; 3 Internal Medicine, St. George's University, St. George, GRD; 4 Internal Medicine, Continental Medical College Lahore, Lahore, PAK; 5 Medicine/Gastroenterology, BronxCare Health System, Bronx, USA

**Keywords:** glucagon-like peptide-1 receptor agonist, liraglutide, exenatide, fibrosis, nonalcoholic steatohepatitis, pathogenesis, semaglutide, non-alcoholic fatty liver disease

## Abstract

Non-alcoholic fatty liver disease (NAFLD) has emerged as one of the lethal causes of chronic liver disease globally. NAFLD can ultimately progress to non-alcoholic steatohepatitis (NASH) given persistent cellular insult. The crux of the problem lies in fat accumulation in the liver, such as increased fatty acid substrates owing to consumption of a high-fat diet, altered gut physiology, and excess adipose tissue. Being the hepatic manifestation of metabolic syndrome, insulin resistance is also among one of the many stimuli. Therefore, drugs, such as glucagon-like peptide-1 receptor agonist (GLP-1 RA) can play a significant role in reducing inflammation, in addition to weight loss and dietary habits. In this review article, we have reviewed the role of exenatide, liraglutide, and semaglutide in the management of NASH. Two of the agents, exenatide and semaglutide, have a predominant role in reducing alanine aminotransferase (ALT) levels, therefore reducing inflammation and promoting weight loss. However, these agents have a lesser impact on the degree of fibrosis. Liraglutide, on the other hand, has been shown to significantly decrease the degree of fibrosis and has been found helpful in reversing mild degrees of steatosis. Therefore, these agents warrant attention to the new perspective that has been presented so that future guidelines may incorporate and streamline individualized therapy.

## Introduction and background

Over the past few decades, non-alcoholic fatty liver disease (NAFLD) has come to the forefront as one of the major causes of chronic liver disease all over the world. It is estimated that around a quarter of the global population has NAFLD [1]. The prevalence of NAFLD is closely linked to the prevalence of type 2 diabetes mellitus (T2DM), obesity, and genetic polymorphisms that increase susceptibility. Globally, the highest numbers of NAFLD have been reported in South America (31%), followed by the Middle East (32%), Asia (27%), the United States of America (USA) (24%), and Europe (23%), with the lowest being in Africa (14%) [2]. In the United States, there is ethnic variability in the prevalence of NAFLD. The highest numbers are among the Hispanic Americans followed by those of European descent and the least in African Americans. This variability can be attributed to genetic factors, socioeconomic conditions, access to health care, and the presence of concurrent diseases such as metabolic syndrome. An interesting observation made by a study was that the prevalence in one ethnic population also varies by country of origin. According to that study, the Hispanics from Mexico have been found to have a higher prevalence of NAFLD than those from the Dominican Republic or Puerto Rico. This sheds light on the important role played by diet, exercise, and alcohol consumption on the pathogenesis of NAFLD [2].

Around one-third of NAFLD progresses to develop into non-alcoholic steatohepatitis (NASH). Hence, around 3% to 5% of the world population is estimated to have NASH [3]. Around 20% of people with NASH progress to cirrhosis. In view of NAFLD’s strong association with obesity and T2DM, NASH is predicted to emerge as the number one cause for liver transplant in the United States [4].

NAFLD can be defined as a condition in which the fat content of the liver is more than 5% and where other causes for hepatic steatosis such as excessive alcohol consumption, medications, infections, and other liver pathologies have been ruled out [5,6]. Alcohol consumption of less than 30 g per day in men and 20 g per day in women are being used to define NAFLD. NAFLD consists of a variety of subsets including non-alcoholic fatty liver, NASH, cirrhosis, and cryptogenic cirrhosis [5]. The steatosis in NAFLD is due to triglyceride accumulation in the hepatocytes. This leads to lobular inflammation, hepatocellular injury, and hepatocyte death. At this stage, the disease acquires a different terminology called NASH. Later, fibrosis with vascular remodeling sets in and pushes the liver into cirrhosis [6].

The etiology of NASH is closely linked with that of NAFLD. NAFLD is thought to be the hepatic manifestation of metabolic syndrome. NAFLD when allowed to persist uncorrected over time progresses to NASH. All the etiologies of NAFLD are linked to abnormalities in fat accumulation in the liver. There are multiple mechanisms for hepatic steatosis such as:

a) Increased delivery of dietary fat to the liver, which could be from increased consumption of fatty food or abnormalities in the gut physiology.

b) Increased availability of free fatty acid from the adipose tissue.

c) Insulin resistance triggered hyperinsulinemia leading to de novo synthesis of lipids [7].

The exact mechanism of progression to NASH is not fully understood but multiple mechanisms have been proposed. However, insulin resistance, oxidative stress, and multiple parallel hits theory have been identified. Parallel hits theory hypothesizes that multiple causes may be simultaneously and not always consecutively acting at the same time to bring about NASH [8]. Once the fat has accumulated in the liver, a sequence of events is set into motion. Steatosis in the liver is usually in the form of triglycerides. Triglyceride as such is not believed to cause much harm to the hepatocytes. When the triglycerides get metabolized, the fatty acids released increases the workload on the endoplasmic reticulum and mitochondria. This causes these organelles to release reactive oxygen species and pro-inflammatory cytokines, which lead to the recruitment of immune cells. A vicious cycle is initiated culminating in hepatocyte injury with ballooning and finally apoptosis. During this cycle, stellate cells in the liver get activated and lay down collagen, causing further remodeling of the liver architecture and fibrosis [7].

The gut-liver axis has been postulated as a cause for NAFLD and NASH. Variations in intestinal permeability can contribute to the development of NAFLD and NASH. A breach in the continuum of the gut epithelial barrier due to loss of intracellular tight junctions paves the way for microbial products such as lipopolysaccharide (LPS) to reach the liver via the portal circulation. In the liver, the LPS will activate toll-like receptors (TLR) on the Kupffer cells, leading to liver damage [9].

Hepatic steatosis has been shown to induce insulin resistance in skeletal, adipose, and hepatic tissue. Insulin resistance due to any cause can be a predictor of the development of hepatic steatosis. In a background of insulin resistance, insulin is unable to inhibit lipolysis in the adipose tissue, leading to an increased supply of fatty acids to the liver for triglyceride synthesis. Furthermore, the enzymes related to de novo lipogenesis get stimulated, thus contributing to the fat accumulation in the liver [10].

In comparison to non-obese individuals, people who are obese were found to have significant differences in the triglyceride metabolism in both adipocytes and hepatocytes. In adipocytes, there is an increased pace of lipolysis, and in hepatic cells, there is an increased release of very-low-density lipoprotein (VLDL) triglycerides into the bloodstream. This increase in the removal of triglycerides from the liver is not sufficient to keep up with the steatosis occurring within the liver. This causes net triglycerides accumulation within the liver [11].

**Figure 1:**
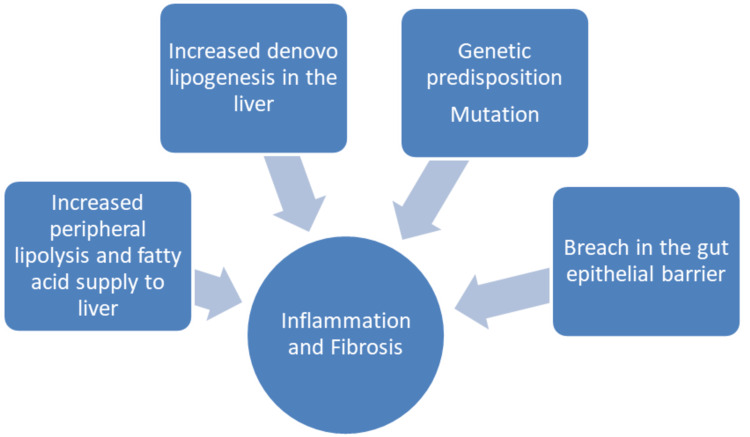
Postulated mechanism for the development of non-alcoholic steatohepatitis

Single-nucleotide polymorphisms involving *PNPLA3* (patatin-like phospholipase domain-containing protein 3) and *TM6SF2* (transmembrane 6 superfamily member 2) are the most well-recognized genetic mutations involved in the development of NASH. *PNPLA3* is linked to an increased risk for the development of fibrosis, and *TMS6F2* controls metabolism and is a predictor of the severity of liver disease in NASH [12]. Possible mechanisms of action behind the development of NASH have been summarized in Figure 1.

There are several risk factors for NASH. They have been summarized in Table 1

**Table 1:** Risk factor for non-alcoholic steatohepatitis

Risk Factors
Obesity
Type 2 diabetes mellitus
Hypertension
Dyslipidemia
Genetic predisposition

Most of the patients with NASH are usually asymptomatic. Common symptoms of NASH are fatigue, dyspepsia, or dull right upper quadrant pain. The majority of the patients with NASH are diagnosed during workup for other unrelated illnesses such as hepatic steatosis in imaging or elevated liver enzymes in routine blood tests. Physical examination in NASH patients is usually unremarkable before the stage of cirrhosis [4].

The invasive modality of liver biopsy is considered the gold standard test for the diagnosis of NASH. Newer non-invasive tests are being researched to facilitate easier and earlier diagnosis of NASH. The non-invasive tests most commonly employed for diagnosis are ultrasound, magnetic resonance imaging (MRI) (sensitivity of 92-100% and specificity of 92-97% for hepatic steatosis) [4], computed tomography (CT), and proton magnetic resonance spectroscopy (quantitatively measures hepatic steatosis) [12]. Transient elastography (FibroScan®) measures the extent of hepatic fibrosis by measuring the stiffness of liver tissue (sensitivity of 85% for advanced fibrosis and 92% for cirrhosis) [4,12]. A few other emerging non-invasive tests and criteria based on fibrogenesis and its markers are being utilized for the diagnosis of NASH [13]. Some of the emerging ones are pro-C3 (procollagen III-direct marker of collagen synthesis (area under the receiver operating characteristic curve [AUROC]: 0.86) [14], NASH NIS4, enhanced liver fibrosis (ELF) test (AUROC: 0.93 in adults) [4], lipidomic serum test, point shear wave elastography (ARFI [acoustic radiation force impulse] for liver stiffness), magnetic resonance elastography (MRE) (sensitivity of 85.4% and specificity of 88.4% in distinguishing F3-F4 from F0-F2) [15], and liver multiscan (maps fibrosis and inflammation; sensitivity of 85% for advanced fibrosis and 92% for cirrhosis; AUROC: 0.85 for F4) [4,14].

Because NASH is affected by the degree of insulin resistance, as suggested by the literature, weight loss and exercise regimens can all lead to increased insulin sensitivity and therefore prevent the development of the disease. Currently, more focus is on anti-diabetic drugs that promote insulin sensitivity.

Glucagon-like peptide-1 receptor agonists (GLP-1 RAs) are a class of antidiabetic agents called incretin mimetics. Incretins are endogenous compounds, including glucagon-like peptide-1 (GLP-1), that improve glycemic control once released into the circulation via the gut. The GLP-1 receptor is a gastrointestinal hormone secreted by the L cells of the intestine and allows for the regulation of blood glucose through glucose-dependent insulin release [16].

GLP-1 is released after the oral ingestion of carbohydrates or fats; It enhances insulin secretion and increases glucose-dependent insulin synthesis and in vivo secretion of insulin from pancreatic beta cells in the presence of elevated glucose [17]. In addition to increases in insulin secretion and synthesis, GLP-1 suppresses glucagon secretion, slows gastric emptying, reduces food intake, and promotes beta-cell proliferation.

GLP-1 agonists have an effect on several tissues including adipose tissues, skeletal muscle, and liver. In adipose tissues, they decrease insulin resistance, thereby reducing the amount of circulating lipotoxic metabolites (non-esterified fatty acids [NEFAs]) and pro-inflammatory mediators [18]. They also have a similar effect on skeletal muscle as they decrease insulin resistance, thus improving uptake of glucose.

**Figure 2:**
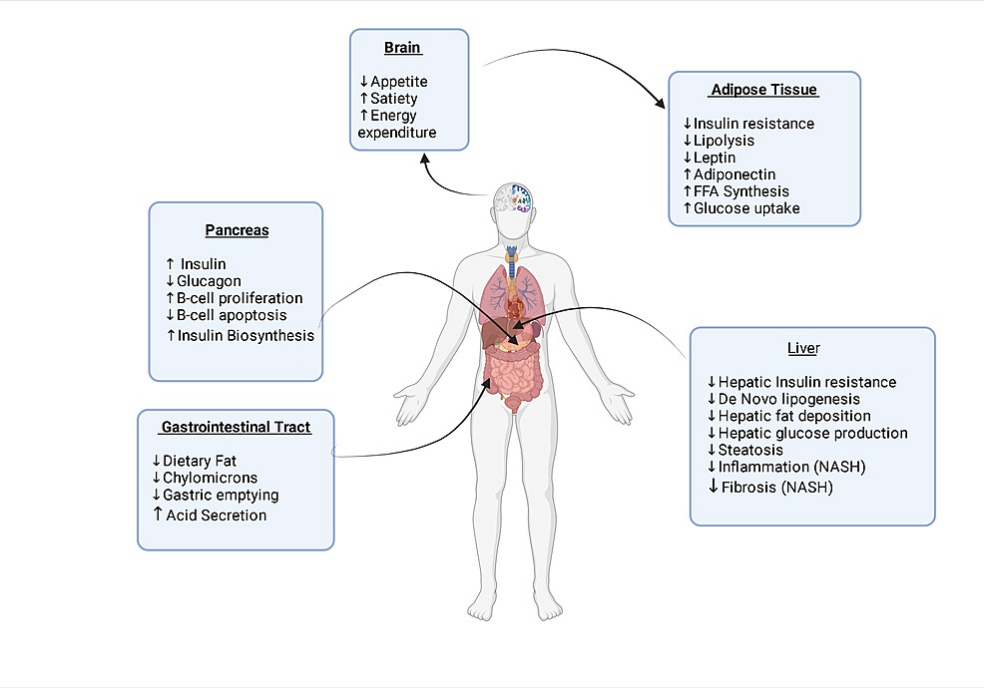
Schematic diagram demonstrating effects of glucagon-like peptide-1 receptor agonist in the management of NASH FFA, free fatty acids; NASH, non-alcoholic steatohepatitis

The mechanism of action of GLP agonists in the management of NASH is likely multifactorial and related to the effect of GLP-1 agonists on adipose tissues, insulin resistance, and the inflammatory process. One mechanism of action is that GLP-1 agonists improved hepatic and adipose insulin sensitivity, thereby reducing the amount of lipotoxic metabolites and pro-inflammatory mediators in the circulation. This reduction in the proinflammatory milieu may explain the beneficial effects of GLP-1 agonists on liver histology [19], especially in the knowledge that persistent inflammation drives fibrosis in NASH. Another mechanism is the reduced hepatic de novo lipogenesis in vivo, which is a key component of hepatic lipid accumulation in NASH [19]; one more potential mechanism is their potential anti-lipogenic action on hepatocytes and restoration of hepatic insulin sensitivity. The role of GLP-1 RA in the management of NASH has been shown in Figure 2.

Now we will review the role of exenatide, liraglutide, and semaglutide in the management of NASH.

## Review

Exenatide

Exenatide is a synthetic analog of exendin-4, a 39-amino-acid agonist of GLP-1 receptor. Exenatide is a short-acting (half-life: <12 hours) GLP-1 RA, which can be given subcutaneously only.

A single-center randomized clinical trial was conducted in China from 2009 to 2010 on a group of 117 patients to investigate the effect of exenatide on blood glucose, body weight, and hepatic enzyme in patients with T2DM and concomitant NAFLD. The patients’ were matched to age and gender groups and further divided into two groups (A and B). In group A, patients (n = 49) were treated with exenatide from week 1 to 4 at 5 μg twice daily and from week 5 to 10 at 10 μg two times a day. In group B (n = 68), patients were treated with metformin at a dose of 0.5 g two times a day. The results revealed that the exenatide group had greater reductions in body weight (4.16 ± 5.32 vs. 1.98 ± 3.28), body mass index (BMI) (1.31 ± 0.98 vs. 0.69 ± 0.94), and aspartate aminotransferase (AST) (7.89 ± 7.8 vs. 75.11 ± 6.98) as compared to the metformin group. Because the inflammatory markers were significantly reduced in the exenatide group, the study concluded that exenatide could help in slowing down the development of NASH [16]. However, this trial had a few limitations. The sample size, for the exenatide group, was particularly small, and this limitation was reflected in the result. In the detailed analysis of the results, it was found that there were lesser patients with NASH in the exenatide group, and therefore a smaller proportion of patients’ liver function reverted to normal. Moreover, an ultrasonographic criterion was used for the diagnosis of NASH rather than histological criteria [16].

Another trial focused on the effect of three years of treatment with exenatide on diabetes, obesity, cardiovascular risk factors, and hepatic biomarkers patients. Patients in this trial were enrolled into one single, open-ended, and open-labeled clinical trial. Patients in this study were randomized into placebo, 5 ug exenatide two times a day, and 10 ug exenatide two times a day for 30 weeks. Patients who completed the 30-week trial were then given the option to continue in the open-label extension of the trial, in which all the patients were given 5 ug exenatide two times a day for four weeks followed by 10 μg of exenatide two times a day for ≥ three years. The randomization was stratified according to HbA1c value (<9.0% and ≥9.0%). This was done to ensure a balanced distribution of the participants in all the groups. Around 217 participants completed three years of treatment with exenatide. Reduction in HbA1C and fasting blood glucose was seen as early as 12 weeks. Around 46% of the patient who completed three years of exenatide achieved an HbA1c of ≤7% and 30% were able to achieve an HbA1c of ≤6.5. A significant weight loss was noticed in patients treated with exenatide. Patients with a BMI of more than 30 kg/m^2^ had a mean loss of -5.8 ± 0.5 kg (p < 0.01), while patients with a BMI of less than 30 kg/m^2^ had a mean loss of -3.9 ± 0.7 kg (p < 0.01). Around 116 participants had an elevated baseline alanine aminotransferase (ALT). Out of these 116 participants, around 41% of the patients were able to achieve normal ALT. More weight loss was seen in patients with elevated ALT at baseline as compared to patients with normal ALT (-6.1 ± 0.6 kg vs. -4.4 ± 0.5 kg; p = 0.03). A 12%, 5%, and 6% decrease in the triglycerides (p = 0.03), total cholesterol (p = 0.07), and low-density lipoprotein cholesterol (p < 0.01), respectively, was seen, while a 24 % increase in high-density lipoprotein cholesterol was seen (p< 0.01). However, the major issue with the study was the absence of a placebo group [20]. Another limitation of the study was the absence of histological examination of the hepatic tissue to confirm the presence of NAFLD at baselines. One of the most common causes of ALT elevation in patient with T2DM is NAFLD. In this study, a decrease in ALT was found to reflect a decrease in liver inflammation.

A single-center study was conducted in China to evaluate the benefit of exenatide treatment on patients with obesity, NAFLD with elevated liver enzymes, and T2DM. A total of 60 patients were randomly divided into the exenatide treatment group and the intensive insulin therapy group. The participants in the exenatide group were given exenatide and insulin glargine, while the participants in the intensive insulin therapy group were given insulin aspart and insulin glargine.

For the first four weeks, the exenatide subgroup was subcutaneously injected with 5 μg exenatide twice daily followed by 10 μg exenatide twice daily for the remaining eight weeks of treatment. The other group was the intensive insulin therapy group (n = 30), which was treated with insulin aspart and insulin glargine. Treatment was continued for 12 weeks in both the groups. After 12 weeks, fasting blood glucose and random blood glucose were significantly decreased in both the groups (p < 0.01); however, there were no significant differences between the two groups (p > 0.05). Bodyweight and waist circumference were significantly decreased in the exenatide group, while an increase in these parameters was seen in the insulin group (p < 0.01). A significant decrease in hepatic injury biomarkers (ALT, AST) and gamma-glutamyl transpeptidase (GGT) was seen in both the arm (p < 0.01). This decrease was more pronounced in the exenatide group as compared to the increased group (p < 0.01). The decrease in hepatic injury biomarkers correlated with the weight loss seen in the exenatide group (p < 0.01). Furthermore, in the exenatide group, ultrasonography revealed that the reversal rate of the fatty liver state was 99.3% as compared to 66.7% seen in the insulin group (p < 0.01). Nausea (20%), vomiting (6.7), and diarrhea (3.3%) were the most common side effect seen in the exenatide group. None of the patients in the exenatide group developed hypoglycemia, while 10% of the insulin group patients developed symptomatic hypoglycemia. None of the patients withdrew from the trial due to drug-related adverse effects [21]. The study's major limitations were the small sample size and cutoffs used for liver dysfunction that created disparity about the extent of side effects and the extent of efficacy.

Liraglutide 

Liraglutide is an acylated GLP-1 agonist derived from human GLP-1 (7-37). While human GLP-1 has only a short half-life of 1.5-2 minutes due to its rapid degradation by the enzyme dipeptidyl peptidase-4 (DPP-4) [22], liraglutide, on the other hand, is a long-acting (half-life: 13 hours) GLP-1 analog with 97% structural homology to the native hormone and is therefore administered as a once-daily subcutaneous injection [23].

It was first licensed in 2009 for glycemic control in obese type 2 diabetic patients. However, given its additional properties of central appetite suppression, delay of gastric emptying time, and induction of dose-dependent weight loss combined with existing evidence of a strong association between metabolic syndrome and NASH [24,25], liraglutide became a promising therapeutic option for consideration in patients with NASH. Initial studies included a meta-analysis involving 4,442 patients assessing the effect of 26 weeks of subcutaneous liraglutide (1.8 mg/day) on liver parameters compared with placebo (LEAD trial). It was found that 26-week liraglutide reduced serum concentrations of hepatic ALT versus placebo (-8.20 vs. -5.01 IU/L; p = 0.03) in T2DM patients, the effects of which were thought to be mediated by its action on improved glycemic control and weight loss [25]. A LEAD-2 sub-study followed up the LEAD trial in the same patient population in which liraglutide 1.8 mg showed a trend toward improving hepatic steatosis versus placebo (liver-to-spleen attenuation ratio: +0.10 vs. 0.00; p = 0.07) [26]. Subsequent studies on liraglutide-treated in vitro murine and human hepatocyte models and in vivo murine models of NASH have shown that liraglutide caused a reduction in liver enzymes concentration, oxidative stress, and hepatic inflammation and steatosis by downexpression of profibrogenic genes (transforming growth factor-beta (TGF-β), collagen type I alpha 1 gene (COL1A1), collagen type I alpha 2 gene (COL1A2), collagen type III alpha 1 chain gene (COL3A1), and hepatic stellate cells activation genes (actin alpha 1, vimentin [VIM]). It was also found to causes a decrease in tissue inhibitor of metalloproteinase 1 (TIMP1), matrix metalloproteinase 13 (MMP13), and inflammatory/immune markers (tumor necrosis factor-alpha, integrin subunit alpha X [ITGAX]) [27,28].

A randomized, multicenter, double-blinded, placebo-controlled phase 2 trial was conducted in four medical centers of the United Kingdom to assess the safety and efficacy of 48 weeks of subcutaneous injections of liraglutide (1.8 mg daily) compared with placebo for patients with biopsy-confirmed NASH (LEAN trial). Eligible patients were randomly assigned to once-daily subcutaneous injections of 1.8 mg liraglutide or placebo groups. A total of nine (39%) of 23 evaluable patients who received liraglutide and underwent end-of-treatment liver biopsy had resolution of definite NASH compared with two (9%) of 22 evaluable patients in the placebo group, thereby meeting the primary outcome (relative risk [RR]: 4.3; 95% CI: 1.0-17.7; p=0.019). Two (9%) of 23 patients in the liraglutide group versus eight (36%) of 22 patients in the placebo group had progression of fibrosis (RR: 0.2; 95% CI: 0.1-1.0; p = 0.04). A more significant proportion of patients in the liraglutide group had histologic improvements in steatosis (83% in the liraglutide group vs. 45% in the placebo group; p = 0.09) and hepatocyte ballooning (61% in the liraglutide group vs. 32% in the placebo group; p = 0.05) compared with the placebo group. However, no differences were seen in lobular inflammation and overall NAFLD activity score among the two groups. No improvement of the fibrosis stage was noted in the liraglutide group despite NASH resolution. Also, three (38%) of eight patients with T2DM and six (40%) of 15 patients without T2DM achieved the primary outcome within the liraglutide treatment group, signifying that histological effects of liraglutide on NASH were not entirely mediated by its action on the improvement of glycemic control. Most of the adverse events noted in the trial were grade 1 (mild) to grade 2 (moderate) in severity, transient, and similar to the two treatment groups for all organ classes and symptoms, except for the incidence of gastrointestinal disorders in 81% of patients in the liraglutide group and 65% of patients in the placebo group, which included diarrhea (38% in the liraglutide group vs. 19% in the placebo group), constipation (27% vs. none), and loss of appetite (31% vs. 8%) [29]. Limitations of the study was that the mean BMI at baseline was significantly different between the liraglutide and placebo groups (34.2 kg/m^2^ [SD: 4.7] vs. 37.7 kg/m^2^ [SD: 62]), suggesting that patients were not matched for weight. Secondly, the study failed to clearly outline the effects of liraglutide on hepatic steatosis in patients with or without weight loss. Despite these limitations, The LEAN study has brought in new insight and given a new therapeutic option in NASH.

Apart from the presence or absence of NAFLD, liraglutide 1.2 mg/day was found to have a significant reduction in liver fat content by 31% (p < 0.01) as assessed by MRI spectroscopy in a trial involving 68 patients with uncontrolled T2DM likely secondary to its weight-lowering effect [30]. Another 26-week, open-label, active-controlled, parallel-group, multicenter trial (Light-On Trial) was conducted in China aiming to evaluate the effects on intrahepatic lipid (IHL), abdominal adiposity, and glycemic control in patients receiving either subcutaneous liraglutide, sitagliptin, or insulin glargine as an add-on treatment to metformin in patients with T2DM with NAFLD. A total of 75 eligible patients with T2DM and NAFLD under inadequate glycemic control by oral metformin were randomized (1:1:1) to receive add-on subcutaneous liraglutide (1.8 mg/day), oral sitagliptin (100 mg/day), or subcutaneous insulin glargine at bedtime (initiated at 0.2 IU/kg/day and titrated to achieve fasting plasma glucose < 7 mmol/L). Results showed that MRI‐PDFF ([magnetic resonance imaging proton density fat fraction] marker for IHL), visceral adipose tissue (VAT), and weight decreased significantly with liraglutide (15.4% ± 5.6% to 12.5% ± 6.4%, p < 0.01; 171.4 ± 27.8 to 150.5 ± 30.8, p = 0.03; 86.6 ± 12.9 kg to 82.9 ± 11.1 kg, p = 0.05, respectively) and sitagliptin (15.5% ± 5.6% to 11.7% ± 5.0%, p = 0.01; 153.4 ± 31.5 to 139.8 ± 27.3, p = 0.027; 88.2 ± 13.6 kg to 86.5 ± 13.2 kg, p = 0.05, respectively). No significant change in MRI‐PDFF, VAT, or body weight was observed with insulin glargine from baseline. Subcutaneous adipose tissue decreased significantly in the liraglutide group (239.9 ± 69.0 to 211.3 ± 76.1; p = 0.020) but not in the sitagliptin and insulin glargine groups. The liraglutide group had significant changes from baseline in MRI‐PDFF (IHL), VAT, and body weight than insulin glargine but did not differ significantly from the sitagliptin group. No significant changes were appreciated in exploratory endpoints involving improvement of NAFLD fibrosis score, FIB-4 (Fibrosis-4) scores, serum prolactin, adiponectin, and IL-6 (interleukin-6) concentrations. The conclusion was that both liraglutide and sitagliptin, but not insulin glargine, reduced body weight, intrahepatic fat, and VAT, in addition to improving glycemic control in patients with T2DM and NAFLD [31].

Semaglutide

Semaglutide is a long-acting (half-life: 24 hours to one month) GLP-1 RA and can be given either orally or subcutaneously. It has around 94% sequence homology with native GLP-1 [32].

A randomized, double-blind, placebo-controlled, multi-national phase 2 trial was conducted to compare the efficacy and safety of three escalating dose levels of subcutaneous semaglutide versus placebo in patients with NASH [33]. The double-blind phase 2 trial further randomly assigned 320 patients into the three interventional arms and corresponding placebo comparators. In this study, patients were randomly assigned to receive either the once-daily semaglutide at a dose of 0.1, 0.2, or 0.4 mg, or the corresponding placebo. The primary endpoint was the resolution of NASH with no worsening of fibrosis. The confirmatory secondary endpoint was an improvement of at least one fibrosis stage with no worsening of NASH. NASH resolution was achieved with no worsening of fibrosis among 40% in the 0.1 mg group (OR: 3.36), 36% in the 0.2 mg group (OR: 2.71), and 59% (OR: 6.87) in the 0.4 mg group, as compared to 17% in the placebo group. There was no significant difference in the improvement of liver fibrosis stage without worsening of NASH between the 0.4 mg semaglutide group and the placebo group (43% vs. 33%; OR: 1.42; p = 0.48). Around 10%, 8%, 5%, and 19% of patients in the 0.1 mg, 0.2 mg, 0.4 mg, and placebo groups, respectively, had worsening of fibrosis. Overall, around 37% of the patients in the 0.4 mg semaglutide group and 15% of the patients in the placebo group both NASH resolution and improvement in fibrosis. A 13% weight loss was seen in the 0.4 mg semaglutide group, while only 1% weight loss was seen in the placebo group. A 58% improvement in serum ALT was seen in the semaglutide 0.4 mg group as compared to 19% improvement in serum ALT in the placebo group. The incidence of nausea, constipation, and vomiting was higher in the 0.4 mg semaglutide group as compared to the placebo group (42% vs. 11%, 22% vs. 12%, and 15% vs. 2%, respectively). In this trial, no significant improvement of the fibrosis stage was seen in the semaglutide group despite NASH resolution and dose-dependent weight loss. One of the reasons suggested for this result was that the trial was not long enough for improvement of fibrosis stage to be seen. Limitations of the study included no implementation of exercise plan and intense diet plan in both the groups, lack of long-term clinical outcomes, racial distribution, and lack of proper documentation regarding alcohol abuse [33].

## Conclusions

NASH occurs due to several various factors that all give rise to stimuli responsible for metabolic syndrome. GLP-1 RAs are one of the newer agents that can combat the problem by reducing ALT levels, thereby decreasing the rate of inflammation and promoting weight loss to such a degree as to reverse steatosis in a few cases. Although steatosis might be reversed, glycemic control using these agents might not relate to the level of reversal. Some agents might also be able to prevent mild degrees of fibrosis from occurring. Therefore, these agents warrant attention for the new perspective that has been presented so that future guidelines may incorporate and streamline individualized therapy.
